# Use of Humic Acids, Zeolite and Bentonite to Mitigate Iron Toxicity and Improve Growth Parameters and Chemical Composition in Maize (*Zea mays* L.)

**DOI:** 10.3390/molecules31111926

**Published:** 2026-06-03

**Authors:** Mirosław Wyszkowski, Natalia Kordala

**Affiliations:** Department of Agricultural and Environmental Chemistry, University of Warmia and Mazury in Olsztyn, Łódzki 4 Sq., 10-727 Olsztyn, Poland; natalia.kordala@uwm.edu.pl

**Keywords:** soil contamination with iron, bentonite, humic acids, zeolite, maize, yield parameters, chemical composition of biomass

## Abstract

Soil contamination with metals is a significant environmental problem that can adversely affect the growth of crops and the quality of yields. Among potentially toxic elements, iron plays a particularly important role, as excessive amounts of it disrupt physiological processes and plant nutrition. The aim of the study was to assess the impact of varying levels of iron contamination in the soil and the effectiveness of selected neutralising substances in mitigating iron stress on the growth, mass, SPAD index and chemical composition of maize biomass. In the absence of additives, excessive iron content (750 mg Fe kg^−1^ of soil) significantly restricted plant growth, causing a 62% reduction in plant height and a reduction in fresh and dry matter mass of 92% and 94%, respectively, compared to the control. However, the lowest dose of iron (250 mg Fe kg^−1^ of soil) in without additions series exhibited a stimulating effect, resulting in an increase in maize height and fresh matter mass. Among the tested neutralising substances, bentonite proved to be the most effective, significantly mitigating the negative effects of iron by increasing plant height by average 92% and fresh and dry matter mass by 167% and 193%, respectively, compared to the series without additives. Bentonite and humic acids also limited the decline in the SPAD index during subsequent growth stages. Soil contamination with iron significantly altered the chemical composition of maize biomass, with the additives used correcting these changes to varying degrees.

## 1. Introduction

Human pressure significantly increases the concentration of trace elements in the soil environment [1]. These elements pose a potentially greater threat to the natural environment and human health than those from natural sources [2]. Among the trace elements accumulating in soils, iron (Fe) occupies a special position due to its dual nature—although it is an essential micronutrient for normal plant growth and development, its excessive concentration leads to serious physiological and biochemical disorders [3].

The main anthropogenic sources of iron in the environment include industrial emissions from metallurgical and smelting plants [4], the combustion of fossil fuels and biomass, and emissions associated with road transport [5]. Agricultural practices, such as the use of mineral fertilisers containing metallic contaminants and sewage sludge, as well as mining and extraction activities generating dust and effluents rich in dissolved forms of iron, also play a significant role in iron accumulation [6]. Excessive availability of iron in the soil environment initiates a series of destructive processes in plant cells. First and foremost, free Fe^2+^ ions catalyse Fenton reactions, leading to the overproduction of reactive oxygen species (ROS) [7], including the hydroxyl radical (•OH) with a very high oxidation potential of 2.8 V [8]. ROS induce chemical modifications and damage proteins (aggregation and denaturation), cause membrane lipid peroxidation and oxidative damage to DNA [9,10]. These changes often lead to irreversible damage to cellular structures and, in extreme cases, to the death of the cell or the entire plant [11]. In chloroplasts, excess iron causes the degradation of photosynthetic pigments, particularly chlorophyll, and photoinhibition of photosystem II (PS II) [10,12]. This leads to a reduction in the quantum efficiency of PS II, and consequently to a reduction in the rate of photosynthesis and yield [13]. In rice grown under flooded conditions, excess iron causes a reduction in grain yield of 12–35% [14].

Excess iron also disrupts cation homeostasis in plant tissues, as Fe^2+^ ions compete for binding sites in transport proteins and for active enzyme sites [15,16]. Suboptimal Fe content in the soil leads to deficiencies in macronutrients, including magnesium, calcium, potassium and phosphorus [17,18], whilst simultaneously increasing sodium uptake [17]. Morphological symptoms of iron toxicity include inhibition of root growth and a reduction in root dry weight [12], as well as a reduction in the number and length of lateral roots [19]. The above-ground parts of plants affected by iron toxicity are characterised by reduced stem height and number of shoots [20], a reduction in leaf area and premature defoliation [21], as well as an inhibition of generative development, which results in delayed flowering and reduced seed weight [22]. The visual symptoms of iron toxicity are primarily the browning of older leaves [23] caused by the accumulation of oxidised polyphenols, spreading from the tip of the leaf to its base [24], chlorosis, mottled necrosis and premature leaf senescence [12]. In some species, the development of young leaves with an intense violet or purple colouration is observed, which is a consequence of the presence of anthocyanins—a group of photoprotective pigments induced under conditions of environmental stress [25].

Most crops, including key cereal species, legumes and vegetables, have not developed effective mechanisms for detoxifying excess iron, making them particularly susceptible to this type of stress [22,26]. The adaptive capacity of plants is further limited by the intensity and duration of Fe^2+^ exposure [18] and co-occurrence of other abiotic stressors, such as drought, salinity or extreme temperatures [27].

In response to the growing problem of soil contamination with trace elements, a variety of remediation methods are being developed, aimed both at immobilising their excess in the soil and at supporting plant tolerance mechanisms. Among remediation techniques, strategies utilising natural aluminosilicates, such as zeolites or bentonites, which, thanks to their three-dimensional crystalline structure, negative charge, and high sorption and ion-exchange capacity, demonstrate the ability to effectively sorb trace element cations from the soil solution [28]. Zeolites constitute a group of naturally occurring hydrated aluminosilicates with a three-dimensional porous structure featuring regular channels and pores, characterised by chemical inertness, a high level of adsorption [29], high cation exchange capacity (CEC; 200–300 meq 100 g^−1^) and a high specific surface area [30]. The mechanism of trace element sorption by zeolites involves the following phenomena: (1) ion exchange of cations that counterbalance the negative charge of the aluminosilicate structure (mainly Na^+^ and Ca^2+^); (2) electrostatic attraction; (3) surface complexation; (4) intrapore adsorption; (5) surface precipitation [31,32].

Bentonite, a clay mineral consisting mainly of montmorillonite (a layered aluminosilicate from the smectite group) is characterised by similar sorption properties, although its adsorption capacity is two to three times lower than that of zeolite, amounting to 80–110 meq 100 g^−1^ [33]. Bentonite acts as a low-permeability barrier and limits the mobility of trace elements through several complementary mechanisms: interlayer cation exchange, surface complexation, electrostatic interactions, and partial inclusion of ions in the interlayer space [34,35]. The positive effects of in situ stabilisation of contaminated soils using bentonite or zeolite were confirmed by Rahimi et al. [36], as well as by Yi et al. [37]. The response of plants to the application of aluminosilicates is strongly influenced by the physicochemical properties of the soil. Aluminosilicates, such as zeolite and bentonite, have a high CEC, enabling them to effectively adsorb not only heavy metals but also macro- and micronutrients. Their use in light, sandy soils can therefore have a beneficial effect on nutrient retention and limit their leaching into the soil profile [38,39]. Conversely, in clay soils, which are characterised by high nutrient retention, high doses of aluminosilicate sorbents can lead to excessive binding of these nutrients and a reduction in their bioavailability to plants, e.g., nitrate nitrogen, readily available phosphorus or potassium [38]. Furthermore, excessive use of bentonite can significantly increase soil pH, leading to the immobilisation of important nutrients in the soil [40]. An important aspect requiring attention when using aluminosilicates is soil pH. In acidic soils (pH < 4.5–5.0), the structure of aluminosilicates becomes destabilised, which can lead to the release of Al^3+^ ions from the sorption complex and an increase in aluminium phytotoxicity [41].

At the same time, remediation methods are being developed that utilise humic substances (HS), which can form stable organometallic complexes with trace element ions, thereby reducing their bioavailability, mobility within the soil profile and phytotoxic potential [42]. The high ability of HS to form complexes with trace elements is attributed to the high surface density of oxygen-rich functional groups (-COOH, -OH, C=O) and, to a lesser extent, functional groups containing nitrogen or sulphur [43]. Among these, carboxyl (-COOH) and phenolic (-OH) groups play a particular role, acting as active centres for the chelation of trace element cations, thereby influencing their speciation and translocation [44,45]. By acting as electron transporters, HS also influence the redox transformation of trace elements, particularly the microbial process, controlling their solubility, mobility and bioavailability [46,47]. The usefulness of humic acids (HA) in controlling the amount of plant-available forms of trace elements in soil was demonstrated by Ondrasek et al. [48]. Importantly, humic acids perform functions that go beyond the immobilisation of toxic trace elements. They also have a positive effect on the physical and biological properties of the soil, stimulate root growth and increase the availability and uptake of nutrients, as well as the concentration of chlorophyll in leaves, which positively affects plant growth and yield [49]. Furthermore, the application of humic acids increases plant resistance to various environmental stressors [50], making them a valuable tool in adapting agriculture to changing climatic conditions.

In view of the above, it was assumed that excessive iron contamination of the soil significantly limits growth, mass, physiological condition (SPAD index) and disrupts the chemical composition of maize biomass, whereas the application of neutralising substances, in particular zeolite, bentonite and humic acids (HA), effectively mitigates the negative effects of iron, although the efficacy of these substances varies and depends on their type and the developmental stage of the plants. The aim of the study was to assess the impact of varying levels of iron contamination in the soil and the effectiveness of selected neutralising substances in mitigating iron stress on growth, mass, the SPAD index and the chemical composition of maize biomass.

## 2. Results

A significant effect was observed of both iron contamination of the soil and the neutralising substances used on plant height, plant biomass, the SPAD leaf greenness index and the content of macronutrients.

### 2.1. Plants Height and Mass

In the series without the use of soil-neutralising substances, iron contamination (as FeCl_3_) with 750 mg Fe kg^−1^ of soil significantly reduced maize growth and mass. Compared with the control, plant height was reduced by 62%, whilst fresh and dry matter mass decreased by 92% and 94% respectively (Table 1). It should also be noted that the lowest iron dose (250 mg kg^−1^ soil) in the without additions series had a significant positive effect on plant height and fresh matter mass.

Among the tested neutralising substances, humic acids and bentonite were the most effective, with bentonite having a markedly stronger effect. Its application limited the negative impact of iron to a maximum of 17% in terms of plant height, 22% for fresh matter mass and 8% for dry matter mass, at the highest contamination level. Compared to the control series, using bentonite resulted in significant increases of 92% in average plant height, and 167% and 193% in fresh and dry matter mass, respectively. Humic acids also improved the analysed parameters, causing significant increases of 24%, 42% and 26%. In contrast, zeolite had a marginal effect on the examined traits.

### 2.2. SPAD Index

**Figure 1:**
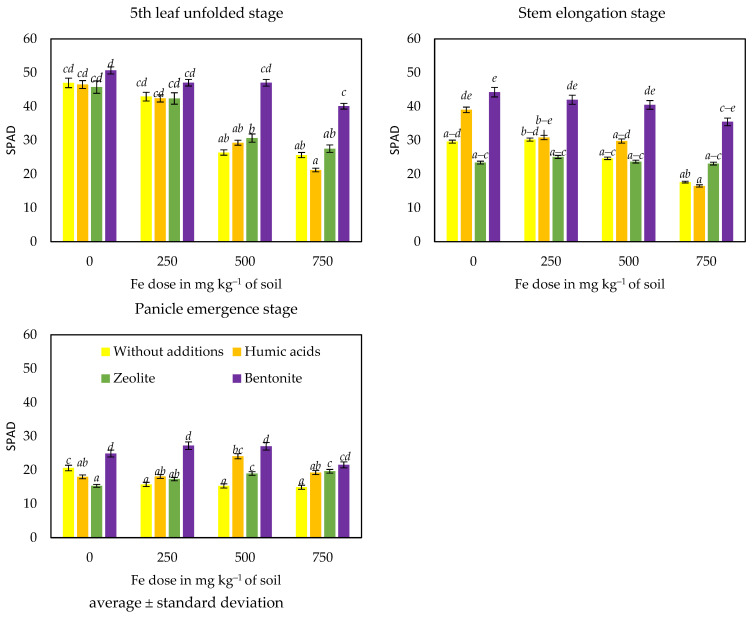
SPAD index in *Zea mays* L. vegetation stages of the 5th leaf unfolded, stem elongation and panicle emergence. Different letters above the figure bars (*a–e*) are significant at *p* ≤ 0.01.

The SPAD index value, which reflects the chlorophyll content in maize leaves, was significantly influenced by the plants’ growth stage, the degree of iron contamination in the soil, and the application of neutralising substances (Figure 1).

In the untreated control series, soil iron contamination (750 mg Fe kg^−1^ of soil) resulted in a significant 46% reduction in the SPAD index at the 5th leaf unfolded stage, and in subsequent growth stages, 41% during the stem elongation stage and 28% during the panicle emergence stage, respectively, compared to the control plot not contaminated with this element. The application of bentonite significantly reduced the negative impact of excessive iron content on the SPAD index in all analysed growth stages. In the series with this additive, the reduction in SPAD values was 21%, 20% and 13% respectively compared to the control (without Fe). As the growing season progressed, a systematic decrease in the SPAD index values in maize leaves was observed, which was associated with the plant ripening. However, it should be noted that the application of bentonite resulted in significantly higher SPAD values, by 30%, 59% and 51% respectively in the subsequent developmental stages, compared to the series without its application. The addition of humic acids also increased this parameter, by 22% during the stem elongation stage and by 19% during the panicle emergence stage. Similarly to the case of maize plant height and mass, the effect of zeolite on the SPAD index was the least pronounced.

### 2.3. Macronutrients

Both iron contamination of the soil and the application of the tested substances significantly altered the chemical composition of maize biomass (Table 2 and Table 3). 

However, the effect of excessive iron content was more pronounced than that of the neutralising additives for most macronutrients. In the series without additives, at the highest level of soil iron contamination (750 mg Fe kg^−1^ of soil), a significant decrease in magnesium content of 27% and phosphorus of 38% was observed, accompanied by significant increases in sodium concentration of 12%, calcium of 23%, potassium by 46% and nitrogen by 63% compared to the uncontaminated plot. However, it should be noted that the lowest dose of iron (250 mg Fe kg^−1^ of soil) caused a few-percentage-point significant increase in calcium content in maize biomass. Application of all the substances studied contributed to a significant reduction in total nitrogen content, by 3% following the application of humic acids, by 16% following the application of zeolite, and by 45% in the case of bentonite. Additionally, bentonite significantly reduced calcium content by an average of 18%, phosphorus by 31%, and potassium by 42%, whilst increasing the accumulation of sodium by 18% and magnesium by 19%. The application of humic acids led to a significant reduction in calcium content by 15%, phosphorus and magnesium content by 19%, while simultaneously increasing sodium concentration by 9%. In contrast zeolite had a relatively minor effect involving a slight, significant reduction in sodium content (by 6%) alongside a significant increase in potassium content (by 8%).

**Table 3:** Sodium, magnesium and calcium content of aerial parts of *Zea mays* L., g kg^−1^ DM.

Fe Dose mg kg^−1^ of Soil	Neutralise Substance				
	Without Additions	Humic Acids	Zeolite	Bentonite	Average
Sodium					
0	0.318 ± 0.010 *^a^*	0.356 ± 0.012 *^cd^*	0.342 ± 0.009 *^bc^*	0.444 ± 0.016 *^f^*	0.365 *^B^*
250	0.320 ± 0.010 *^ab^*	0.355 ± 0.012 *^cd^*	0.308 ± 0.008 *^a^*	0.410 ± 0.015 *^e^*	0.348 *^A^*
500	0.350 ± 0.011 *^cd^*	0.348 ± 0.013 *^c^*	0.309 ± 0.008 *^a^*	0.360 ± 0.014 *^cd^*	0.342 *^A^*
750	0.357 ± 0.011 *^cd^*	0.401 ± 0.014 *^e^*	0.306 ± 0.007 *^a^*	0.371 ± 0.015 *^d^*	0.359 *^B^*
Average	0.336 *^B^*	0.365 *^C^*	0.316 *^A^*	0.396 *^D^*	0.353
*r*	0.941	0.675	−0.808	−0.903	−0.307
Magnesium					
0	1.892 ± 0.061 *^ef^*	1.526 ± 0.053 *^c^*	1.973 ± 0.043 *^fg^*	1.779 ± 0.061 *^d^*	1.792 *^B^*
250	1.797 ± 0.058 *^de^*	1.508 ± 0.053 *^c^*	1.903 ± 0.042 *^ef^*	2.032 ± 0.071 *^g^*	1.810 *^B^*
500	1.711 ± 0.055 *^d^*	1.376 ± 0.049 *^b^*	1.737 ± 0.036 *^d^*	2.267 ± 0.081 *^h^*	1.773 *^B^*
750	1.388 ± 0.046 *^b^*	1.110 ± 0.038 *^a^*	1.385 ± 0.030 *^b^*	2.000 ± 0.070 *^fg^*	1.471 *^A^*
Average	1.697 *^B^*	1.380 *^A^*	1.750 *^D^*	2.019 *^C^*	1.711
*r*	−0.943	−0.928	−0.950	0.580	−0.803
Calcium					
0	4.535 ± 0.145 *^b^*	3.350 ± 0.117 *^a^*	4.889 ± 0.108 *^bc^*	3.245 ± 0.130 *^a^*	4.005 *^A^*
250	6.533 ± 0.199 *^e^*	4.810 ± 0.158 *^bc^*	5.103 ± 0.112 *^cd^*	3.185 ± 0.107 *^a^*	4.908 *^B^*
500	5.589 ± 0.179 *^d^*	5.153 ± 0.170 *^cd^*	6.564 ± 0.144 *^e^*	5.502 ± 0.185 *^d^*	5.702 *^C^*
750	5.594 ± 0.175 *^d^*	5.633 ± 0.187 *^d^*	6.212 ± 0.117 *^e^*	6.230 ± 0.162 *^e^*	5.917 *^D^*
Average	5.563 *^B^*	4.737 *^A^*	5.692 *^B^*	4.541 *^A^*	5.133
*r*	0.353	0.944	0.854	0.933	0.971

Average ± standard deviation; *r*—coefficient of correlation between the Fe dose and the tested parameters. Different letters to the right of the values (*a–h* and *A–D*) are significant at *p* ≤ 0.01.

### 2.4. Relations Beetwen Variables

**Figure 2:**
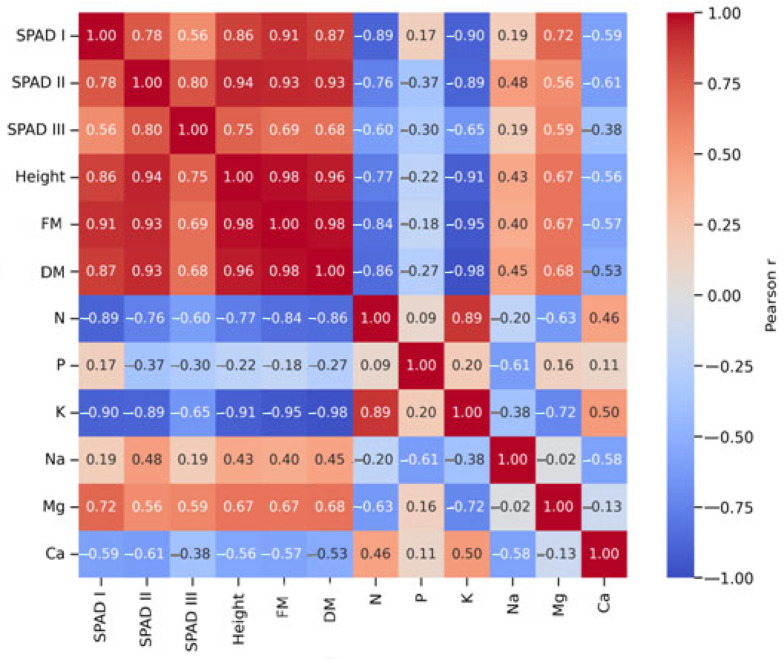
Correlation between variables in *Zea mays* L. Explanations: SPAD I—SPAD at the 5th leaf unfolded stage, SPAD II—SPAD at the stem elongation stage, SPAD III—SPAD at the panicle emergence stage; FM—fresh matter biomass, DM—dry matter biomass.

**Figure 3:**
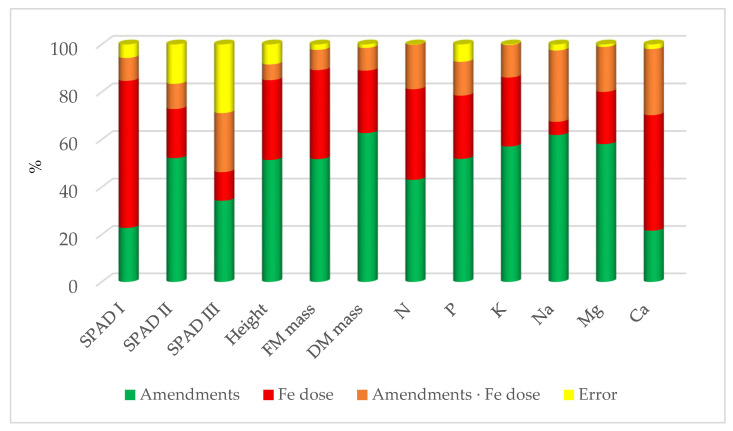
Structure of the factors influencing maize biomass variability (percent). Explanations: SPAD I—SPAD at the 5th leaf unfolded stage, SPAD II—SPAD at the stem elongation stage, SPAD III—SPAD at the panicle emergence stage; FM—fresh matter, DM—dry matter.

The relationships between the indicators under study were determined by calculating correlation coefficients (Figure 2), and the extent of the impact of iron contamination in the soil and the application of neutralising substances was expressed as a percentage (Figure 3).

The Pearson correlation heatmap illustrates the interrelationships between plant physiological indicators (SPAD I–III), growth and mass traits (plant height, fresh matter yield—FM yield, dry matter yield—DM yield) and macronutrient content (N, P, K, Na, Mg, Ca). The use of a uniform colour scale allows for the unambiguous identification of both strong positive correlations and weaker or negative relationships between the variables under study.

The highest significant correlation coefficients were observed between yield-related traits and plant growth. Plant height showed a strong positive correlation with both fresh matter yield (FM yield) and dry matter yield (DM yield), indicating a close relationship between growth intensity and biomass productivity. Similarly, an equally strong significant positive correlation was found between FM yield and DM yield, confirming that both indicators describe a consistent yield gradient.

Very strong positive and significant correlations were also observed between SPAD I, SPAD II and SPAD III, confirming the high repeatability and stability of the SPAD index across successive measurement dates. High SPAD values were significantly and positively correlated with growth and yield traits, indicating the co-occurrence of an intensive assimilation apparatus with higher plant biomass.

Nitrogen content showed positive and significant correlations with plant height and both yield indices, placing this element in the group of variables associated with the main productivity gradient. A similar, though slightly weaker, pattern of correlations was observed for phosphorus and potassium, which were also positively correlated with yield-related traits. In the case of calcium, the significant correlations with growth and yield parameters were moderate. Meanwhile, magnesium and sodium showed weaker or more varied significant relationships with the other variables. Their position on the heat map indicates a different nature of variability compared to elements directly linked to growth and yield intensity.

The layout of the heat map clearly indicates the existence of two main clusters of variables: (1) a group comprising SPAD I–III, plant height and FM and DM yields, characterised by strong positive internal correlations, and (2) a group of macronutrients, in which N, P and K show a stronger association with yield-related traits, whilst Na and Mg are less strongly correlated with this gradient of variation.

The analysis of the contribution of sources of variation shown in Figure 3 revealed that amendments were the dominant factor significantly influencing most of the studied indicators, particularly DM mass (62.74%), Na (61.93%), Mg (58.10%), K (57.10%), P (51.91%), fresh matter mass (51.83%), plant height (51.42%) and SPAD II (52.22%). These indicators were characterised by a dominant contribution of amendments in explaining the variation, indicating that the type of soil amendment applied was of key importance in determining mass, plant growth and the accumulation of selected macronutrients. The Fe dose was the main factor significantly differentiating SPAD I (61.87%) and Ca (48.65%), and also showed a significant contribution to the variability of N (38.11%), fresh matter mass (37.37%) and plant height (33.56%). For these parameters, the iron dose was the main factor differentiating plant response, particularly evident in relation to initial SPAD measurements and calcium content, and also had a significant effect on N and growth parameters. A clear effect of the amendments × Fe dose interaction was found for Na (29.97%), Ca (27.80%) and SPAD III (24.83%), as well as for Mg (18.90%), N (18.71%), P (14.22%) and K (13.68%), indicating the varied and complex nature of the response of these indicators to the interaction of both factors.

## 3. Discussion

### 3.1. Effect of Iron Contamination on Maize Growth and SPAD Values

In our own studies, soil contamination with iron at the highest dose (750 mg Fe kg^−1^ soil) resulted in significant disturbances in maize development, manifested by a reduction in the SPAD index, plant height, and fresh and dry matter mass. Similar observations were reported by Ahmed et al. [11], who, under conditions of excessive exposure of rice to Fe^2+^ (300 mg L^−1^), noted a reduction in total chlorophyll content (by 22%), the membrane stability index (by 17%), above-ground biomass mass (by 26%) and net photosynthesis rate (by 30%) compared to the control (uncontaminated) sample. In a subsequent experiment, the same authors [23] demonstrated that rice plants begin to show symptoms of toxicity at Fe concentrations of 600 and 900 mg L^−1^. Under these conditions, most parameters related to growth, biomass and grain mass were reduced, particularly root dry matter (by 15%), above-ground biomass dry matter (by 14%), number of panicles per plant (by 15%) and grain mass (by 10%), relative to the control. A reduction in shoot height (by 56%) and root length (by 28%) in *Catharanthus roseus* grown under conditions of Fe excess (50 mM) compared to the control was also reported by Tisarum et al. [12]. The fresh and dry matters of shoots and roots were also reduced, by over 50% and 60% respectively. Furthermore, the authors demonstrated that under these conditions, the SPAD index decreased by 15% and the net photosynthetic rate by 83%. Excess iron leads to chlorophyll degradation and photoinhibition of PS II [51], which is reflected in reduced SPAD values observed in the present study. This effect may be attributed to the inhibition of 5-aminolevulinic acid synthesis, a precursor of chlorophyll, or an increase in the activity of the chlorophyll-degrading enzyme—chlorophyllase [52]. Furthermore, Fe may inhibit protochlorophyllide reductase, a key enzyme in the biosynthesis pathway of photosynthetic pigments [53,54]. Xu et al. [55] demonstrated that exposure of *Pisum sativum* L. to Fe^2+^ concentrations exceeding 200 µM led to a reduction in stomatal conductance, chlorophyll content and deactivation of the PS II reaction centre, as well as an increase in the content of unsaturated fatty acids in chloroplast membranes, which reduced the energy efficiency of photosynthesis. Consequently, the rate of net photosynthesis was reduced, adversely affecting plant growth and dry matter. The particularly pronounced inhibition of root growth under conditions of iron excess results from direct contact between the root tip and external Fe in the soil solution [56]. Excess Fe inhibits taproot growth by reducing both elongation and cell division [57]. A reduction in the root meristem in *Arabidopsis thaliana* in response to excess Fe was demonstrated by Reyt et al. [58]. The authors attributed this phenomenon to the arrest of the cell cycle via the ROS-activated SMR5/SMR7 cyclin-dependent kinase inhibitor pathway. O’Leary et al. [59] observed that a Fe^2+^ concentration of 600 µM in the medium leads to the accumulation of callose in the roots, predominantly in the phloem, which blocks plasmodesmata and disrupts symplastic transport, further limiting elongation growth. In turn, Zhang et al. [56] demonstrated in studies on *Arabidopsis thaliana* that excess iron (>300 μM) induces the accumulation of nitric oxide (NO) in the root apical zone, leading to the loss of K^+^ via non-selective cation channels, causing loss of cell turgor, growth inhibition and cell death.

### 3.2. Macronutrient Content in Maize Biomass Under Iron Stress Conditions

The disturbances in macronutrient uptake observed in the present study reflect both ion competition at the level of membrane transport proteins [60] and the morphological consequences of iron-induced root damage [61], as previously described. In our own studies, above-optimal iron content resulted in disturbances in macronutrient levels, leading to a significant reduction in Mg and P content and the accumulation of Na, Ca, K and N in the above-ground biomass of maize. Partially similar results were obtained by Majerus et al. [62], who noted a reduction in Ca (by 40%), K (by 56%), Mg (by 38%) and P (by 31%) in the leaf sheaths of rice (*Oryza glaberrima*) grown hydroponically with an excess of iron (500 mg L^−1^), compared to the control object. The authors attributed the significant reduction in phosphorus uptake to the formation of insoluble Fe-P complexes in the rhizosphere and on the root surface. The reduction in Mg content in the above-ground biomass of maize observed in our own studies is due to ionic antagonism between Fe and Mg. Excess iron in the soil competitively inhibits the uptake of Mg^2+^ ions by shared membrane transporters with broad substrate specificity, reducing its availability to the plant [63]. In turn, Aung et al. [64] demonstrated that under conditions of excessive Fe content, genes encoding Ca transporters are repressed. These results are consistent with our previous observations [65], further confirming the disruptive effect of iron excess on macronutrient homeostasis in maize. The increase in the content of N, Ca, K and Na in the above-ground biomass of maize exposed to excess iron, as observed in our own studies, may be the result of what is known as element concentration. When biomass accumulation is severely inhibited, the pool of absorbed nutrients, although it may be lower in absolute terms, is distributed over a smaller tissue mass, resulting in an increase in their concentration per unit of dry matter. With regard to nitrogen, the effect of iron on enzymatic activity may play an additional role. There are reports that moderate iron stress may stimulate nitrate reductase activity and temporarily increase NO_3_^−^ assimilation in plant tissues [66], which could partly explain the observed increase in the content of this element. Çelik et al. [67] demonstrated a reduction in K, N and P content in both the leaves and roots of maize grown under conditions of iron excess (120 µM). Similarly, Mehraban et al. [68] demonstrated a reduction in potassium content in the shoots and roots of rice grown under conditions of Fe excess (>250 mg L^−1^). Under these conditions, the authors also noted an intensification of oxidative stress and an increase in ROS levels. These phenomena may be the cause of disturbances in potassium homeostasis. Overproduction of reactive oxygen species leads to the inactivation (oxidation) of transport enzymes and the peroxidation of membrane lipids, resulting in a loss of membrane integrity and a reduction in their barrier function. Consequently, there is an uncontrolled outflow of potassium ions from the cells into the apoplastic space [69]. Under conditions of severe oxidative stress, an increase in the expression levels of the *SKOR* (Stellar K^+^ Outward Rectifier) and *GORK* (Guard cell Outwardly Rectifying K^+^) genes is also observed; these genes encode potassium channels that facilitate K^+^ efflux, which further exacerbates the deficit in functional potassium uptake and transport systems [70]. ROS-activated K^+^ efflux via GORK channels causes a drastic loss of K^+^ from plant cells, which stimulates proteases and endonucleases and promotes programmed cell death [71]. The results obtained in this study regarding the K, Ca and N content in the above-ground biomass of maize grown on iron-contaminated soil do not unequivocally confirm the findings of other authors who observed an antagonistic effect of excess Fe on the uptake of monovalent and divalent cations. These discrepancies can be interpreted in the context of iron speciation in the soil, its pH and organic matter content, the developmental stage of the plants at the time of biomass harvest, or the species-specific tolerance of maize.

The use of FeCl_3_ as a source of contamination introduces both Fe^3+^ and Cl^−^ ions into the soil, which could theoretically complicate the interpretation of the results. Cl^−^ ions were introduced into the soil in a single application prior to sowing and, being highly mobile in the soil solution and not subject to specific adsorption by soil particles [72], were subject to leaching and gradual dilution within the soil profile during the growing season, which reduced their availability to roots. In contrast, Fe^3+^ ions are strongly retained in the soil through adsorption, precipitation as hydroxides and oxyhydroxides, and complexation with organic matter [73], retaining their phytotoxic potential throughout the duration of the experiment. Maize (*Zea mays* L.) belongs to species that are moderately tolerant to salinity and Cl^−^ ions [74], and the key mechanism of this tolerance is the restriction of Cl^−^ translocation from roots to shoots. Zhang et al. [72] demonstrated that high soil Cl^−^ concentrations (757.1 mg kg^−1^ DM) did not affect the fresh and dry above-ground biomass of maize, its growth or leaf water content. Only a slight reduction in the SPAD index was observed, without any inhibition of the rate of photosynthesis. The dose-dependent reductions in fresh and dry matter yield, the decrease in SPAD values, and the disturbances in macronutrient uptake observed in our own studies are consistent with the well-documented symptoms of iron toxicity, including stunted stem growth, browning of leaf tips, impaired phosphate uptake, and interference with the absorption of Ca^2+^, Mg^2+^ and K^+^ [23,24,62]. This pattern clearly differs from the stress response characteristic of chloride toxicity or general salinity, which manifests primarily as necrosis of leaf tips, stomatal closure and a reduction in tissue water potential [75]. Indirect evidence for the limited phytotoxicity of Cl^−^ ions is also provided by studies on other species. Hütsch et al. [76] demonstrated that the potato varieties Marabel and Désirée can be fertilised with KCl instead of K_2_SO_4_ without reducing tuber yield or impairing their quality.

### 3.3. Neutralising Substances as a Tool for Reducing Iron Phytotoxicity—Impact on Maize Growth and Chemical Composition

In our own studies, the application of humic acids, bentonite and zeolite to iron-contaminated soil limited the negative impact of excess Fe on growth, mass, SPAD index and the chemical composition of maize biomass. The best results were obtained in the series with bentonite and humic acids. The effect of zeolite on the studied traits was marginal.

#### 3.3.1. Humic Acids

Compared to the series without additives, the application of humic acids resulted in a 24% increase in average plant height and a 42% and 26% increase in fresh and dry matter mass, respectively. The content of total nitrogen, as well as phosphorus, calcium and magnesium, was reduced, whilst the concentration of sodium increased. Similar trends were observed in our previous study [65], which focused on the energy potential of maize biomass used as a phytostabiliser—HA application positively affected dry matter mass and plant height, while simultaneously reducing macronutrient content in the above-ground biomass. These observations are consistent with the research by Eyheraguibel et al. [77], who, despite the clearly beneficial effect of humus-like substances (HLS) on maize biomass (an 85% increase), also observed a reduction in the content of K, Mg, S and P in the plant stems. This seemingly contradictory result can be explained by the ‘dilution effect’; the significant increase in maize dry matter mass (by 26% in our study) was not proportionally offset by increased nutrient uptake, which consequently led to a reduction in their content within plant tissues. Partially similar results were obtained by Dinçsoy and Sönmez [78], who, after applying HA at a rate of 40 kg da^−1^, recorded an increase in wheat mass and grain mass, but also an increase in the content of K, Ca and Mg in the straw. Aşık et al. [79] demonstrated in wheat studies that soil application of humic substances (1 g kg^−1^ soil) resulted in an 19% increase in dry matter and a significant increase in the uptake of calcium (by 30%), magnesium (by 35%), nitrogen (by 36%), phosphorus (by 41%) and potassium (by 58%), compared to the control. The increased uptake of macronutrients may be linked to the stimulating effect of humic substances on the expression of transporter proteins for these components [80]. Furthermore, HA, demonstrating the ability to adsorb exchangeable cations such as Mg, Ca and K, prevents their leaching by percolating water, thereby increasing the availability of these elements to plants [81]. In a field experiment conducted by Li et al. [82], the application of humic acids at a rate of 250 kg ha^−1^ resulted in a significant increase in maize mass (by 30%), plant height (by 15%), above-ground dry matter (by 14%) and leaf area index (by 15%) compared to the control (without HA application). The mechanism of this effect may be related to the direct influence of humic acids on plant metabolism through the stimulation of membrane H^+^-ATPase activity, which improves nutrient uptake as well as plant growth and development [83]. Furthermore, by binding with soil minerals, HA forms an organic-inorganic complex, improving the availability of nutrients such as nitrogen, phosphorus and potassium in the soil [78,84]. Humic substances also act as auxin-like growth regulators, stimulating root system development and increasing the root’s absorption surface area [80]. Under conditions of iron excess, this effect is particularly beneficial, as it enables plants to more efficiently uptake water and nutrients from the soil volume not contaminated with this element. Roudgarneyad et al. [85] demonstrated that foliar application of humic acids (300 mg L^−1^ ha^−1^) increased the chlorophyll a and b content in field bean leaves, which directly translates into improved photosynthetic efficiency and biomass growth. In the context of iron toxicity, which leads to chlorophyll degradation via the overproduction of ROS [51], the protective effect of humic acids may be linked to their antioxidant properties [86] and their ability to chelate excess Fe, thereby limiting its involvement in the Fenton reaction. This is confirmed by the present study, in which the SPAD index value increased in the series with HA addition by 22% during the stem elongation stage and by 19% during the panicle emergence stage. The positive effect of humic substances on the SPAD value may also result from improved availability of nitrogen and magnesium (key components of the chlorophyll molecule) [87] and a reduction in the toxic effect of iron on enzymes in the chlorophyll biosynthesis pathway [52].

#### 3.3.2. Bentonite

In our own studies, the application of bentonite had a positive effect on various physiological traits of maize grown under iron-deficient conditions, including plant height, fresh and dry matter mass, and the SPAD index. This positive effect of bentonite on maize growth observed in this study is consistent with its well-documented ability to immobilise Fe^2+^ via ion exchange and surface adsorption [88], and to buffer soil pH—both of which limit Fe^2+^ solubility and bioavailability [34,89]. Similar observations were reported by Farghali and Tammam [90], who, following the application of a 5% bentonite amendment to the soil, noted an increase in both the length of maize shoots and roots, as well as their fresh matter. In a field experiment conducted by Al-Zayadi and Hussein [91], the application of bentonite at a rate of 20 Mg ha^−1^ to the soil resulted in a significant improvement in the growth parameters of sorghum (*Sorghum bicolor*), including plant height (an increase of 23%), leaf area (an increase of 31%) and chlorophyll content (an increase of 29%) compared to the control plots. Under these conditions, the authors also noted an increase in biomass mass (by 57%), grain mass (by 23%) and 1000-grain weight (by 18%). In our own studies, the introduction of bentonite into Fe-contaminated soil resulted in a reduction in N content (by 45%), K (by 42%), P (by 31%), and Ca, Na and Mg (by 18–19%) in the above-ground parts of maize compared to the uncontaminated control. Partially similar results were obtained by Wyszkowski and Ziółkowska [92], who demonstrated that bentonite applied at a rate of 2% of soil matter significantly modified the macroelement content in the above-ground biomass of oats (*Avena sativa* L.). The authors observed the strongest effect in the case of sodium, the content of which increased by 125% compared to the control. Similarly, the potassium content increased by 13%, and the phosphorus content by 19%. In contrast, the magnesium and calcium content in the above-ground biomass of oats decreased by 25% and 14%, respectively. An increase in the nitrogen, phosphorus and potassium content in the leaves of *Catharanthus roseus* L. following the application of bentonite to the soil at a rate of 6 g L^−1^ was reported by Shahad and Hamid [93]. Similar results were obtained by Youssef [94], who demonstrated that the application of bentonite significantly increased the N, P and K content in both the foliage and tubers of potatoes. Conversely, a different trend was observed for sodium, whose concentration in plant tissues decreased as the bentonite dose increased. The mechanisms through which bentonite improves plant growth and mass are multifaceted and relate both to the improvement of the physical and chemical properties of the soil and to its direct impact on plant physiological processes. Thanks to its water-retention capacity and high cation exchange capacity, bentonite facilitates water uptake by roots and increases the availability of nutrients to plants [95], which, under abiotic stress conditions, is crucial for maintaining plant cellular homeostasis. Improvements in soil physical properties include a reduction in bulk density, an increase in water-holding capacity, and an improvement in aggregate structure. In terms of chemical properties, however, the application of bentonite to the soil contributes to pH neutralisation, a reduction in electrical conductivity, an increase in CEC, and improved availability of N, P and K. These changes stimulate the internal physiological and biochemical processes of plants, leading to increased mass and improved quality of its components [91]. Long-term studies conducted by Czaban and Siebielec [96] showed that the application of bentonite to poor sandy soils (at rates of 3, 6 and 12 kg m^−2^) significantly modifies their chemical properties and the availability of macronutrients, even 38 years after the mineral was applied. The application of bentonite at the highest dose resulted in a 70% increase in soil sorption capacity compared to the control soil, an increase in pH from 5.9 to 8.3–8.9, and higher levels of K^+^, Ca^2+^ and Mg^2+^, which may explain the increase in magnesium content in maize tissues observed in this study.

#### 3.3.3. Zeolite

The results obtained indicate only a marginal positive effect of zeolite on maize growth and mass, the SPAD index value, and the chemical composition of the plants, which is partly at odds with reports by numerous authors indicating a marked improvement in agronomic and nutritional parameters following the application of this mineral [97,98]. These discrepancies are likely due to methodological differences, including the type of substrate, the form and source of the contaminant, and the duration of the experiment. It is also worth noting that the effectiveness of zeolite is strongly dependent on its dosage, mineralogical type and particle size; these parameters vary between individual studies in a way that precludes direct comparisons. However, the results obtained by Danish et al. [99] provide a certain analogy to our research; in a pot experiment with maize under glyphosate-induced stress, they found that the application of zeolite at a 5% dose improved all analysed plant growth parameters (plant height, root length, fresh and dry above-ground biomass, SPAD value and photosynthetic rate), but without accompanying changes in the N, K and P content of maize leaves. The results presented by Amirahmadi et al. [98], however, were fundamentally different. The authors, investigating the effect of zeolite on maize growth in cadmium-contaminated soil (30 mg kg^−1^ soil), demonstrated that its addition at a dose of 5 g kg^−1^ increased plant height by 74%, dry above-ground biomass by 84%, and root dry matter by 78% compared to the control (without zeolite). At the same time, the researchers noted a significant increase in potassium content (by 20%), phosphorus (by 47%) and nitrogen (by 71%) in the leaves, as well as an improvement in soil properties, expressed as a 52% increase in CEC and pH (7.65 versus 7.25), and an accumulation of potassium, phosphorus, carbon and nitrogen, by 11%, 75% 124% and 443%, respectively. According to the authors, the protective effect of zeolite resulted from an ion exchange process in which Cd^2+^ ions from the soil solution were exchanged for K^+^, Ca^2+^ and Mg^2+^ ions trapped within the zeolite structure, which simultaneously reduced cadmium toxicity and improved the mineral nutrition of the plants. The scale of the observed effects in that case was significantly greater than in the present experiment, which may result both from differences in the type of used contaminant (Cd vs. Fe) and from the differing reactivity of zeolite towards the indicated elements in the soil environment. The effect of zeolite on the content of macronutrients in plant tissues is not unidirectional, a finding also confirmed by other reports in the literature. Adamczyk-Mucha et al. [100] demonstrated that the application of zeolite to the soil at a rate of 5% contributed to an increase in the phosphorus and magnesium content in lettuce leaves, whilst simultaneously reducing the calcium content, compared to the control. In contrast, the nitrogen and potassium content, as well as the sodium and sulphur content, remained largely unchanged. A similarly ambiguous effect of zeolite on the chemical composition of spring barley biomass grown in cobalt-contaminated soil was noted by Kosiorek and Wyszkowski [101]. Among the macronutrients analysed, sodium showed the most pronounced and unambiguous response, with its average content in shoots and roots increasing by 143% and 78%, respectively, compared to the control. The authors attributed this phenomenon to the ion-exchange properties of clinoptilolite, which, by releasing Na^+^ cations from the silicate lattice during exchange with other cations, increases the availability of sodium to plants. The phosphorus content also increased in both the shoots (by 35%) and roots (by 9%) of barley, whilst the potassium content decreased slightly in both organs studied, and the nitrogen and magnesium contents did not undergo significant changes. The mechanism of zeolite’s beneficial effect on nutrient management in the soil is linked to its crystalline structure, which enables the adsorption of cations, including NH_4_^+^, K^+^ and Ca^2+^, thereby promoting both nutrient retention and limiting their loss through leaching [102]. In this context, Sepaskhah and Barzegar [103] attributed the higher rice grain mass in plots treated with zeolite precisely to increased nitrogen retention in the soil and its more even distribution within the soil profile. Zeolites can also act as a slow-release source of macronutrients, particularly potassium [104], which are released as a result of ion exchange processes with cations present in the soil solution [105]. This may explain the increase in K content (by 8%) in the above-ground biomass of maize observed in this study.

### 3.4. Limitations of the Study and Practical Implications

The results of this study clearly indicate the potential of humic acids and bentonite to mitigate iron toxicity and improve growth and mass parameters in maize grown in soils contaminated with this metal. However, in order to develop comprehensive, cost-effective and environmentally sustainable remediation strategies, long-term field studies are required, taking into account diverse soil and climatic conditions, varying levels of iron contamination, and the optimisation of application rates and frequencies for these soil amendments.

This study was conducted under controlled pot experiment conditions, which is an advantage but also a significant limitation when it comes to extrapolating the results to field conditions. Pot experiments, whilst essential for identifying mechanisms of action, are characterised by a limited soil volume (9 kg of soil per pot), varying water-air ratios within the soil profile, a lack of natural spatial variability in soil properties, and simplified thermal and hydrological conditions compared to a field ecosystem. These factors may intensify phytotoxic effects or alter the efficacy of the applied remediation materials relative to real-world conditions.

Another limitation is the developmental stage at which the plants were harvested. The maize was harvested during the panicle emergence stage (BBCH 59), i.e., at the time of intensive vegetative growth and the onset of the generative phase, immediately prior to flowering. The choice of this harvest date was justified—the BBCH 59 stage is the period during which the plant reaches its maximum above-ground vegetative biomass. Harvesting at this stage therefore allowed for the assessment of the cumulative effects of contamination and remediation on total biomass and final chemical composition. However, this did not allow for the tracking of changes across subsequent developmental stages, during which plants may exhibit varying sensitivity to iron stress. The rate at which arable crops take up trace elements during the later stages of the growing season may vary due to increases in biomass (dry matter), a reduction in the availability of pollutants in the soil, or the transition of plants from vegetative to reproductive growth [106]. Future studies should include multiple sampling during the growing season (e.g., BBCH 16, BBCH 19, BBCH 51, BBCH 70) to better characterise the dynamics of changes in macronutrient uptake, the effectiveness of the sorbents used, and the impact of soil iron contamination on mass and the mineral composition of the grain.

## 4. Materials and Methods

### 4.1. Soil Preparation

The research was conducted as a pot experiment under controlled conditions in a vegetation hall and focused on analysing the combined effects of two experimental factors. The principal factor involved soil enrichment with iron at four levels: 0, 250, 500, and 750 mg kg^−1^ of soil, applied in the form of iron(III) chloride (FeCl_3_). The secondary factor consisted of soil amendments, namely humic acids (HA), zeolite, and bentonite. Humic acids were incorporated at a dose of 0.9 g kg^−1^ soil, whereas zeolite and bentonite were added at 15 g kg^−1^ soil, corresponding to 1.5% of soil mass. Humic acids were applied twice during the experimental period: initially at the time of pot preparation and subsequently at the 5th leaf unfolded (BBCH 15—Biologische Bundesanstalt, Bundessortenamt and Chemical Scale), stage of the test plant. The optimal doses of substrates were selected based on previously performed experiments [65].

The soil substrate was collected from the topsoil (ornithic–humus horizon) of a light soil classified as loamy sand according to granulometric composition [107]. The soil was characterized by an acidic reaction, with a pH (1 M KCl) of 4.56. Their hydrolytic acidity reached 28.25 mmol(+) kg^−1^, whereas the total exchangeable bases amounted to 45.33 mmol(+) kg^−1^. Consequently, the cation exchange capacity was determined at 73.58 mmol(+) kg^−1^, with a base saturation level of 60.69%. In terms of organic matter and nutrient status, the soils contained 4.38 g kg^−1^ of total organic carbon and 0.537 g kg^−1^ of total nitrogen, both expressed on a dry matter basis. The availability of macronutrients was moderate, with concentrations of available phosphorus, potassium, and magnesium equal to 74.80 mg P kg^−1^, 122.34 mg K kg^−1^, and 40.46 mg Mg kg^−1^ DM, respectively. Notably, the iron content was relatively high, reaching 8991 mg Fe kg^−1^ DM. The chemical composition of the applied materials showed clear differences in macroelement content. Zeolite was characterized by moderate concentrations of nutrients, containing 1.84 g P, 5.44 g K, 0.55 g Mg, 15.85 g Ca, and 0.22 g Na kg^−1^ DM, along with 4.92 g Fe kg^−1^ DM. In contrast, bentonite exhibited lower levels of phosphorus and potassium (0.47 g P and 2.43 g K kg^−1^ DM), but was richer in magnesium, calcium, and sodium, reaching 5.03 g Mg, 26.72 g Ca, and 12.11 g Na kg^−1^ DM. Calcium oxide was distinguished by a very high calcium content (347.99 g kg^−1^ DM), accompanied by smaller amounts of magnesium (2.65 g kg^−1^ DM), potassium (0.77 g kg^−1^ DM), phosphorus (0.10 g kg^−1^ DM), sodium (0.07 g kg^−1^ DM), and 4.24 g Fe kg^−1^ DM. Humic acids were introduced in the form of an organo-mineral fertilizer with a high content of bioactive compounds. This material contained 250 g kg^−1^ humic acids, 220 g kg^−1^ organic carbon, as well as 100 g kg^−1^ amino acids and 100 g kg^−1^ betaine. The nitrogen concentration reached 40 g kg^−1^, while the converted macronutrient contents were 0.44 g P, 41.5 g K, and 3.02 g Mg kg^−1^. Additionally, the preparation supplied B vitamins (3 mg kg^−1^ of B_1_ and 95 mg kg^−1^ of B_2_) and was characterized by a total organic matter content of 520 g kg^−1^.

At the beginning of the experiment, all treatments received identical doses of essential macronutrients to ensure adequate plant nutrition, amounting to 160 mg N, 54 mg P, and 170 mg K per kg of soil. These nutrients were supplied as CO(NH_2_)_2_, KH_2_PO_4_, a mixture of KH_2_PO_4_ and KCl, and MgSO_4_·7H_2_O. FeCl_3_ and soil substrates were added simultaneously during the setup of the experiment. The soil, together with iron, amendments, and fertilisers, was thoroughly homogenised, and 9 kg portions were placed into polyethylene pots (dimensions of 25 cm for height, 24 cm for bottom diameter of the pot and 19 cm for upper diameter of the pot).

### 4.2. Plant Growth Parameters

Plants were sown the next day after the experiment was established. Maize (*Zea mays* L.) cultivar Lokata was selected as the phytoremediation species due to its agronomic importance, high biomass production, and considerable nutrient uptake capacity. The maize cultivar Lokata belongs to the FAO maturity group 220, which classifies it as an early-maturing hybrid. Each experimental treatment was established in three replicates, with six plants cultivated per pot. The pots were arranged in the vegetation hall in a completely randomized design, and their positions were periodically rearranged to minimize the effects of spatial heterogeneity. Soil moisture was kept constant (60% of the maximum water capacity) throughout the growing period by regular watering with redistilled water. Climatic conditions during the experimental period showed noticeable monthly variability. The highest relative air humidity was recorded in April (72%), followed by July (67%) and June (62%), whereas the lowest value occurred in May (55%). Insolation increased markedly from 188.7 h in April to a peak of 318.7 h in May, remaining relatively high in June (301.8 h) before slightly decreasing to 275.5 h in July. Air temperature exhibited a gradual upward trend over the study period, rising from 7.8 °C in April to 13.1 °C in May, and reaching 17.9 °C and 18.7 °C in June and July, respectively.

Harvesting was performed after panicle emergence (BBCH 59) on the 75th day after sowing the seeds. At this stage the representative plant samples were collected for subsequent laboratory analyses.

### 4.3. Analytical Methodologies

Leaf greenness was evaluated using the SPAD index at three developmental stages: the 5th leaf unfolded (BBCH 15), the stem elongation stage (BBCH 30), and during panicle emergence (BBCH 59). Leaf greenness was assessed using the SPAD index, measured with a Konica Minolta SPAD-502Plus chlorophyll meter (Konica Minolta, Inc., Chiyoda, Japan) [108]. SPAD measurements were performed on three replicates per plant, with readings taken from fully developed leaves (6 plants per pot). The leaf greenness index values (SPAD, Soil Plant Analysis Development) obtained using the SPAD 502Plus chlorophyll meter are a direct indicator of the relative chlorophyll content in the leaf. The chlorophyll meter is equipped with two different light sources: 650 nm (corresponding to the maximum absorption by chlorophyll a and b) and 940 nm (corresponding to the farred spectrum, which is absorbed by other components of the leaf tissue structure). This is used to correct the result calculated by the microprocessor and displayed in conventional units on the screen. Many authors demonstrate a close correlation between SPAD readings and analytical measurements of chlorophyll content [109,110], as well as nitrogen content [111] and crop yield levels [112,113,114]. The SPAD index may therefore serve as a good indicator of proper plant development [115]. In the context of our study, SPAD measurements enabled a non-invasive, rapid assessment of the physiological condition of maize under conditions of stress induced by excess iron and in response to the applied soil amendments.

At harvest, the plants were cut at ground level and separated as above-ground biomass. Immediately after harvesting, the fresh weight was recorded using an analytical balance. The plant material was then dried in a forced-air oven at 60 °C until a constant weight was reached. After drying, the samples were weighed again to determine the dry weight. Dry matter mass was calculated based on the difference between fresh and dry weight and expressed per pot. The final results were expressed in g per pot. Plant material was finely chopped and subsequently milled. The processed samples were stored in polyethylene containers prior to chemical analysis. For macronutrient determination, plant material was subjected to wet digestion using sulphuric acid. Total nitrogen content was quantified according to the Kjeldahl procedure [116,117]. Phosphorus concentration was determined colorimetrically, whereas potassium, calcium, magnesium, and sodium were analysed by atomic absorption and emission spectrometry [118].

### 4.4. Statistical Methods

Statistical analysis of the results was carried out using Statistica version 13.3 [119]. A two-way analysis of variance (ANOVA) was used to assess the influence of experimental factors, and the significance of differences between means was verified using Tukey’s (HSD) test. The normality of data distribution was assessed using the Shapiro–Wilk test, while the homogeneity of variance was evaluated with Levene’s test. A heat map was also produced, showing Pearson’s correlation coefficients between the indicators studied in maize, including plant growth parameters, physiological indicators (SPAD index), and the content of macroelements and selected ions in plant biomass (N, P, K, Ca, Mg, and Na). The percentage of observed variance was calculated using the eta-squared (η^2^) method within the two-way ANOVA, which enabled the estimation of the proportion of total variation attributed to individual factors: the degree of iron contamination, the remediation materials, and their interaction. All calculations were performed at a statistical significance level of *p* ≤ 0.01, ensuring high reliability of the conclusions drawn.

## 5. Conclusions

Excess iron in soil adversely affects key functional traits of maize, including plant growth, biomass production, physiological status, and mineral composition, confirming the species’ high sensitivity to iron-induced stress. The stimulatory response observed at low iron levels indicates a biphasic effect, with iron acting as both an essential micronutrient and a phytotoxic factor depending on its concentration.

Among the tested amendments, bentonite demonstrated the highest effectiveness in alleviating iron stress, promoting the recovery of growth-related traits and improving physiological performance, as reflected by enhanced chlorophyll status (SPAD index). This confirms its practical relevance as a soil amendment for mitigating iron toxicity.

Iron contamination significantly disrupts the ionic balance of maize, leading to decreased accumulation of phosphorus and magnesium, alongside increased uptake of nitrogen, potassium, calcium, and sodium. Such changes indicate a profound disturbance in nutrient homeostasis under excess iron conditions. The application of neutralising substances, particularly bentonite, partially restored this balance by moderating the uptake and accumulation of these elements, thereby improving the nutritional status of plants.

Overall, the results highlight that effective mitigation of iron toxicity requires not only limiting its direct phytotoxic effects but also stabilising plant mineral nutrition. The demonstrated influence of soil amendments on morphological, physiological, and biochemical properties underscores their importance in maintaining crop productivity under metal stress. Further research should focus on their long-term effects on nutrient dynamics, iron bioavailability, and agronomic performance under field conditions across diverse soils and cropping systems.

## Figures and Tables

**Table 1:** Height, fresh matter (FM) and dry matter (DM) mass of aerial parts of *Zea mays* L.

Fe Dose mg kg^−1^ of Soil	Neutralise Substance				
	Without Additions	Humic Acids	Zeolite	Bentonite	Average
Height (cm)					
0	100.7 ± 3.2 *^c–e^*	122.9 ± 4.2 *^d–g^*	96.3 ± 2.1 *^c–e^*	165.8 ± 6.6 *^g^*	121.4 *^C^*
250	107.6 ± 3.1 *^c–f^*	126.5 ± 3.8 *^e–g^*	101.3 ± 2.2 *^c–e^*	155.5 ± 5.2 *^g^*	122.7 *^C^*
500	68.1 ± 2.2 *^a–c^*	103.1 ± 3.6 *^c–f^*	80.4 ± 1.8 *^b–d^*	146.3 ± 4.9 *^fg^*	99.5 *^B^*
750	38.2 ± 1.2 *^ab^*	36.1 ± 1.3 *^a^*	43.9 ± 1.2 *^ab^*	137.6 ± 4.5 *^e–g^*	64.0 *^A^*
Average	78.6 *^A^*	97.2 *^B^*	80.5 *^A^*	151.3 *^C^*	101.9
*r*	−0.916	−0.873	−0.886	−0.999	−0.920
Aerial parts fresh matter mass (g plant^−1^)					
0	65.27 ± 2.09 *^de^*	92.73 ± 3.25 *^g^*	53.57 ± 1.18 *^cd^*	116.25 ± 4.65 *^i^*	81.95 *^C^*
250	70.57 ± 2.26 *^d–f^*	83.48 ± 2.55 *^e–g^*	66.70 ± 1.47 *^de^*	108.45 ± 4.28 *^hi^*	82.30 *^C^*
500	18.05 ± 0.58 *^ab^*	43.93 ± 1.54 *^c^*	37.15 ± 0.82 *^bc^*	108.50 ± 4.34 *^hi^*	51.91 *^B^*
750	5.17 ± 0.17 *^a^*	6.33 ± 0.22 *^a^*	9.28 ± 0.20 *^a^*	90.88 ± 3.52 *^f–h^*	27.92 *^A^*
Average	39.76 *^A^*	56.62 *^C^*	41.68 *^B^*	106.02 *^C^*	61.02
*r*	−0.911	−0.973	−0.847	−0.914	−0.946
Aerial parts dry matter mass (g plant^−1^)					
0	13.00 ± 0.42 *^ef^*	16.25 ± 0.57 *^f^*	8.53 ± 0.19 *^cd^*	23.13 ± 0.93 *^h^*	15.23 *^C^*
250	12.97 ± 0.41 *^ef^*	13.30 ± 0.45 *^ef^*	10.47 ± 0.21 *^de^*	21.03 ± 0.74 *^gh^*	14.44 *^C^*
500	2.32 ± 0.07 *^ab^*	6.28 ± 0.22 *^c^*	5.37 ± 0.12 *^bc^*	19.68 ± 0.79 *^g^*	8.41 *^B^*
750	0.78 ± 0.03 *^a^*	0.85 ± 0.03 *^a^*	1.42 ± 0.03 *^a^*	21.37 ± 0.65 *^gh^*	6.10 *^A^*
Average	7.27 *^A^*	9.17 *^B^*	6.45 *^A^*	21.30 *^C^*	11.05
*r*	−0.921	−0.989	−0.863	−0.606	−0.961

Average ± standard deviation; *r*—coefficient of correlation between the Fe dose and the tested parameters. Different letters to the right of the values (*a–i* and *A–C*) are significant at *p* ≤ 0.01.

**Table 2:** Nitrogen, phosphorus and potassium content of aerial parts of *Zea mays* L., g kg^−1^ DM.

Fe Dose mg kg^−1^ of Soil	Neutralise Substance				
	Without Additions	Humic Acids	Zeolite	Bentonite	Average
Nitrogen					
0	13.30 ± 0.42 *^e^*	12.09 ± 0.42 *^d^*	13.21 ± 0.29 *^e^*	9.29 ± 0.35 *^a^*	11.97 *^A^*
250	14.79 ± 0.47 *^f^*	14.70 ± 0.51 *^f^*	15.63 ± 0.34 *^g^*	10.50 ± 0.42 *^b^*	13.91 *^B^*
500	25.99 ± 0.83 *^L^*	22.45 ± 0.79 *^j^*	19.55 ± 0.43 *^h^*	11.34 ± 0.43 *^c^*	19.83 *^D^*
750	21.70 ± 0.69 *^i^*	24.13 ± 0.81 *^e^*	14.98 ± 0.35 *^f^*	10.97 ± 0.41 *^bc^*	17.94 *^C^*
Average	18.95 *^D^*	18.34 *^C^*	15.84 *^B^*	10.52 *^A^*	15.91
*r*	0.789	0.968	0.446	0.850	0.853
Phosphorus					
0	3.446 ± 0.110 *^f^*	2.205 ± 0.077 *^b^*	3.213 ± 0.071 *^ef^*	2.053 ± 0.080 *^b^*	2.730 *^C^*
250	2.991 ± 0.096 *^d–f^*	2.435 ± 0.085 *^bc^*	3.058 ± 0.068 *^d–f^*	1.984 ± 0.079 *^ab^*	2.617 *^C^*
500	2.519 ± 0.071 *^b–d^*	2.133 ± 0.075 *^b^*	2.895 ± 0.062 *^c–e^*	2.123 ± 0.083 *^b^*	2.417 *^B^*
750	2.139 ± 0.059 *^b^*	2.209 ± 0.077 *^b^*	2.209 ± 0.051 *^b^*	1.450 ± 0.056 *^a^*	2.002 *^A^*
Average	2.774 *^A^*	2.246 *^B^*	2.844 *^C^*	1.903 *^C^*	2.441
*r*	−0.999	−0.286	−0.926	−0.703	−0.961
Potassium					
0	13.71 ± 0.43 *^c^*	12.41 ± 0.41 *^b^*	16.43 ± 0.36 *^e^*	9.51 ± 0.38 *^a^*	13.02 *^A^*
250	13.79 ± 0.44 *^c^*	14.83 ± 0.51 *^d^*	16.79 ± 0.37 *^e^*	10.33 ± 0.41 *^a^*	13.93 *^B^*
500	21.52 ± 0.70 *^h^*	20.87 ± 0.69 *^gh^*	20.05 ± 0.44 *^fg^*	9.69 ± 0.39 *^a^*	18.03 *^C^*
750	20.00 ± 0.66 *^f^*	23.20 ± 0.81 *^i^*	21.35 ± 0.45 *^h^*	10.28 ± 0.40 *^a^*	18.71 *^D^*
Average	17.25 *^B^*	17.83 *^C^*	18.65 *^D^*	9.96 *^A^*	15.92
*r*	0.839	0.982	0.959	0.520	0.954

Average ± standard deviation; *r*—coefficient of correlation between the Fe dose and the tested parameters. Different letters to the right of the values (*a–i* and *A–D*) are significant at *p* ≤ 0.01.

## Data Availability

The data are contained within the article.
